# Symptomatic bone marrow metastasis in triple-negative breast cancer: a case report and literature review

**DOI:** 10.3389/fonc.2025.1570355

**Published:** 2025-03-28

**Authors:** Chenhui Zheng, Jingjing Dai, Yu Wang, Danni Zheng, Renzhi Lin, Zhibao Zheng

**Affiliations:** ^1^ Department of Surgical Oncology, Taizhou Central Hospital (Taizhou University Hospital), Taizhou, China; ^2^ Department of Pathology, Taizhou Central Hospital (Taizhou University Hospital), Taizhou, China

**Keywords:** symptomatic bone marrow metastasis, triple-negative breast cancer (TNBC), advanced breast cancer, case report, thrombocytopenia, nutritional support

## Abstract

Bone metastasis is common in breast cancer, but symptomatic bone marrow involvement is exceedingly rare. This report presents a case of a young female patient with triple-negative breast cancer (TNBC) initially presenting with symptomatic bone marrow metastasis. Upon admission, blood tests revealed severe thrombocytopenia. Bone marrow biopsy confirmed tumor cell infiltration, and immunohistochemistry identified metastatic breast cancer cells. The patient received paclitaxel and eribulin chemotherapy combined with transfusion support, achieving significant symptom control. This rare case highlights the importance of early detection and aggressive management in TNBC with bone marrow metastasis.

## Introduction

Breast cancer is one of the most prevalent malignancies among women worldwide. Advances in diagnosis and treatment have significantly improved survival rates; however, approximately 20–30% of early-stage breast cancer patients develop metastases (1), with nearly 90% of these cases resulting in mortality (2). The most common sites of metastasis include bone, liver, and lung (3). Bone marrow metastasis (BMM) represents a rare yet highly aggressive condition, often manifesting as hematologic abnormalities such as anemia and thrombocytopenia. While up to 68.8% of metastatic breast cancer patients develop bone metastases, many of which involve micrometastases to the bone marrow (3), symptomatic BMM is exceedingly rare, accounting for only 2.1% of cases (4). Due to the limited number of reported cases and poor prognosis, the clinical characteristics and therapeutic implications of symptomatic BMM remain poorly understood. This report aims to contribute to the existing literature by presenting a case of symptomatic BMM in breast cancer, providing additional clinical insights into this rare and challenging condition.

## Case report

**Figure 1:**
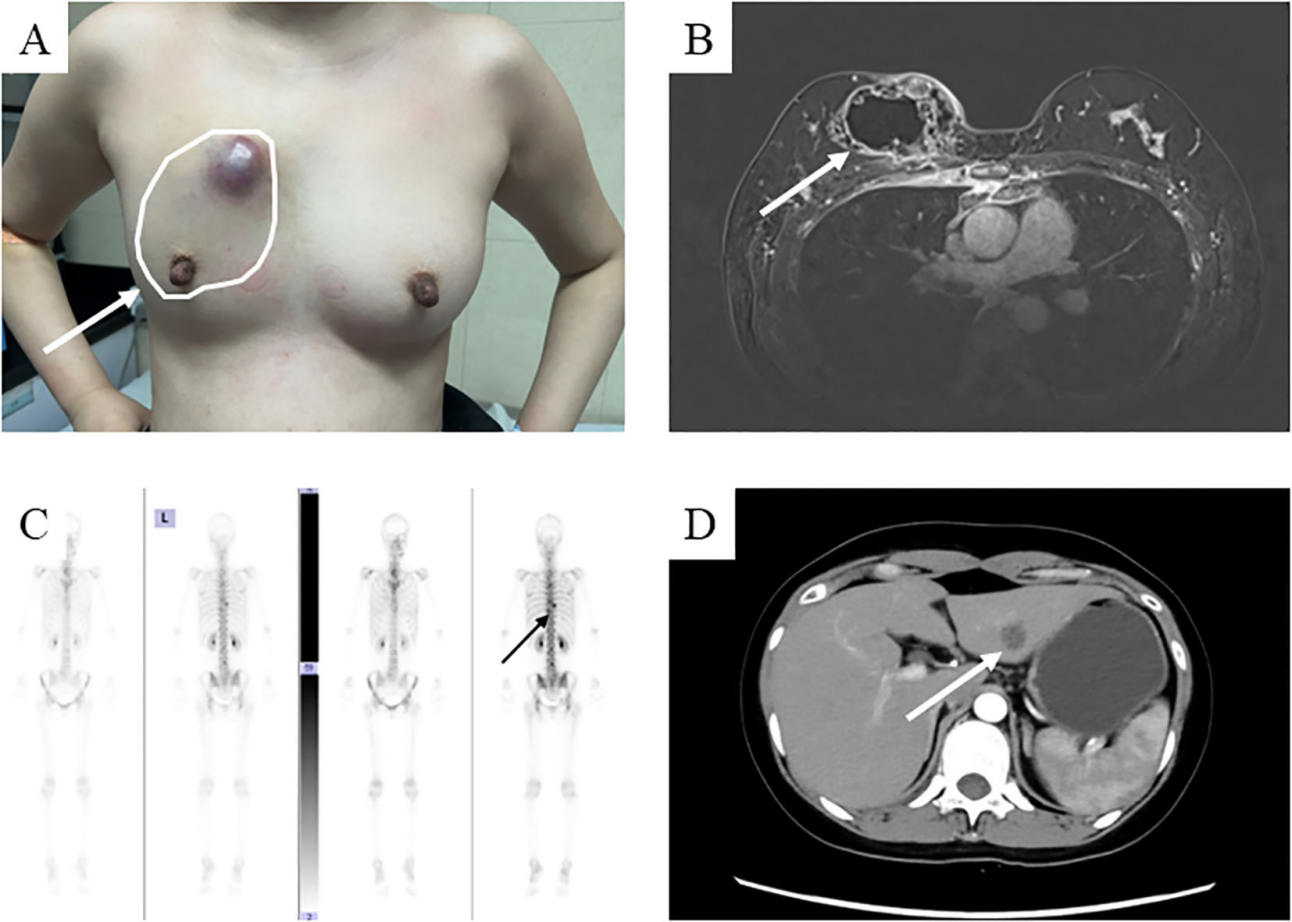
The condition of the patient at the time of their initial visit. **(A)** A hard mass measuring 10x8 cm with unclear borders and poor mobility is present in the right breast, accompanied by localized skin protrusion and redness; **(B)** The breast mass is shown on the enhanced magnetic resonance imaging scan. **(C)** Enhanced Computed tomography images scan shows liver metastasis; **(D)** Whole Body Scan suggests metastatic lesions at the 5th, 8th, and 9th thoracic vertebrae.

**Figure 2:**
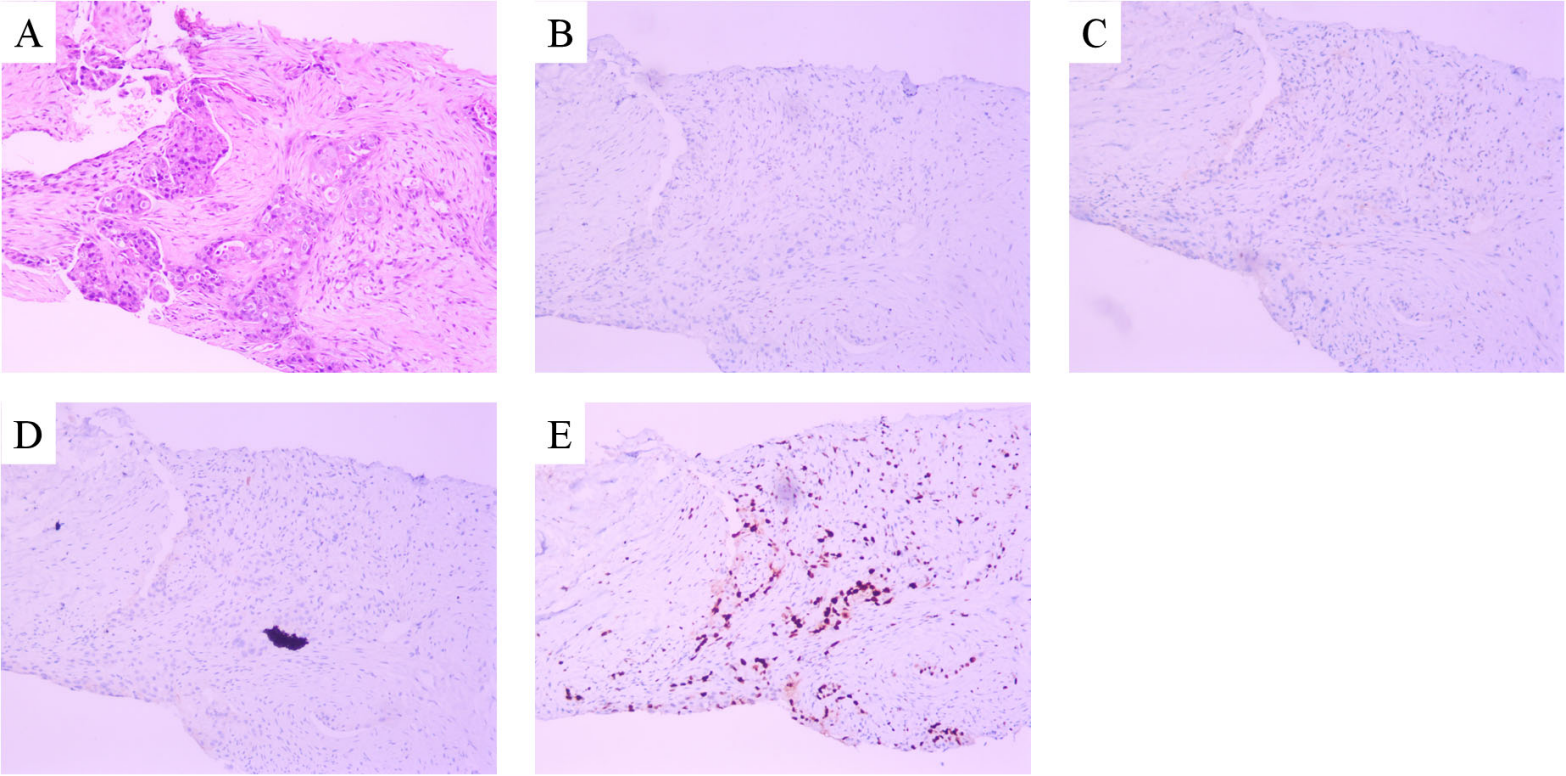
Microscopy examination of the breast specimen. **(A)** Hematoxylin and eosin(HE) staining suggested invasive ductal carcinoma of breast (original magnification, 100 ×); The immunohistochemical staining of ER (negative, **B**), PR (negative, **C**), c-erbB2 (negative, **D**) and Ki-67 proliferation index (50% positive, **E**).

**Figure 3:**
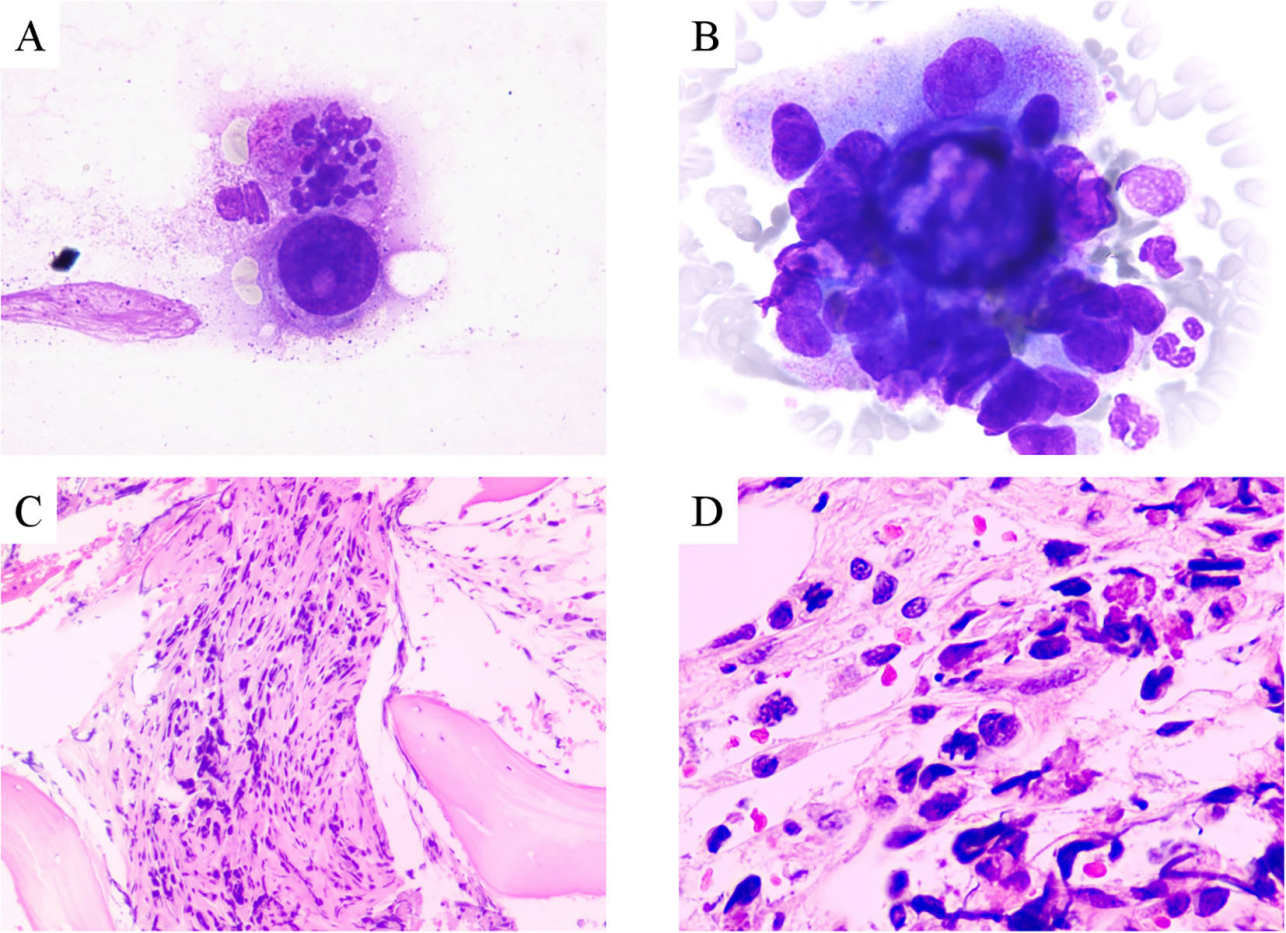
**(A–B)** Adenocarcinoma nests in bone marrow aspiration smears (Wright-Giemsa stain, 1000x); Metastatic adenocarcinoma in bone marrow (HE stain, **(C)** 100x; **(D)** 400x).

**Figure 4:**
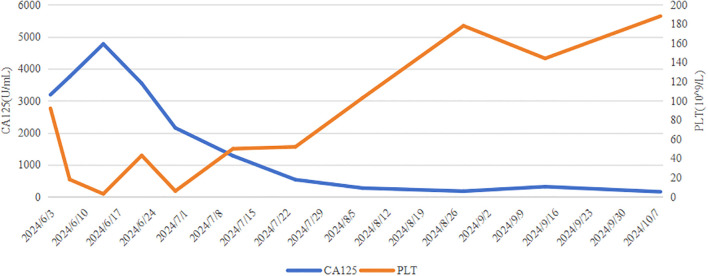
Changes in breast cancer tumor CA125 and platelet count.

A 39-year-old Asian woman with no prior medical conditions or family history of cancer, presented with a right breast lump and skin redness. Her physical exam revealed a 10 cm x 10 cm breast mass with redness and enlarged axillary lymph nodes (**Figure 1A**). A core needle biopsy of the right breast tumor revealed invasive ductal carcinoma, with negative ER, PR, and HER2 (**Figures 2A–E**). A subsequent needle biopsy of axillary lymph nodes confirmed breast cancer metastasis (**Supplementary Figures 1A–C**). Magnetic Resonance Imaging revealed a mass in the right breast, located in the upper inner quadrant. Contrast-enhanced imaging showed significant heterogeneous enhancement, suggestive of malignancy (**Figure 1B**), The systematic assessment indicates osteolytic changes in the vertebral bodies (T5, T8, T9)(**Figure 1C**), consistent with vertebral metastasis. CT scans also reveal multiple low-density liver nodules with ring enhancement, supporting the diagnosis of liver metastasis (**Figure 1D**). On the patient’s initial visit to our hospital on June 3, 2024, a routine blood examination revealed a platelet count of 92 × 10^9/L. A follow-up platelet count, taken only four days later, suddenly plummeted to 18 × 10^9/L. A bone marrow biopsy performed following hematology consultation revealed infiltration by breast cancer cells (**Figure 3**). Immunohistochemistry was positive for CK-pan and CK-7, and negative for ER, PR, HER2, CK-20 and Mum-1. BRCA1/2 mutation analysis was negative, with a PD-L1 CPS score of 3. Treatment began on June 10, 2024. The patient was treated with a paclitaxel regimen (paclitaxel 120mg ivgtt qw), followed by denosumab for bone metastatic disease. For thrombocytopenia management, when the platelet count was less than 30× 10^9/L, 10 units of platelets were transfused, and thrombopoietin receptor agonists, specifically hetrombopag and romiplostim, were also utilized. Following the completion of 12 cycles of paclitaxel-based chemotherapy, the breast mass was reduced to approximately 4 × 4 cm, accompanied by significant skin retraction. During the continued administration of chemotherapy, platelet counts progressively increased, reflecting recovery of bone marrow function. Concurrently, CA125 levels exhibited a gradual decline, indicating effective suppression of tumor activity and reduction in tumor burden by the therapeutic regimen (**Figure 4**). However, during continued paclitaxel therapy, the breast mass enlarged to 5 × 5 cm, meeting RECIST 1.1 criteria for progressive disease (PD). Due to suspected paclitaxel resistance, treatment was switched to eribulin (eribulin 1.9mg d1d8 ivgtt q3w). Following four cycles, the breast mass decreased to 3 × 3 cm, with slight reduction in liver metastases but no significant change in bone lesions (**Supplementary Figures 2A–C**). Unfortunately, due to inadequate nutritional support during treatment, the patient experienced a decline in body weight from 63 kg to 45 kg over a six-month therapeutic course by January 2025, culminating in severe malnutrition and refeeding syndrome. This complication ultimately precluded further tolerance of chemotherapy. After consultation with the nutrition team, she is currently receiving aggressive nutritional rehabilitation. She contacted in February 2025 and reported a stable condition.

## Discussion

This case report involved a young woman with TNBC who presented with significant thrombocytopenia at diagnosis. Differential diagnoses included immune thrombocytopenic purpura and hypersplenism. Initial platelet count was 92 × 10^9/L, indicating thrombocytopenia and a need to evaluate for bone marrow involvement. After only four days, platelet levels decreased to 18 × 10^9/L, necessitating bone marrow aspiration. Immunohistochemistry confirmed metastasis of breast cancer to the bone marrow. Cytokeratin markers such as CK7 and CK20 assist in determining the origin of tumor cells in the bone marrow. CK7 is widely expressed in breast, lung, and ovarian adenocarcinomas, while CK20 is primarily expressed in gastric, colonic, and pancreatic tumors (5). This case underscores the importance of considering bone marrow aspiration in breast cancer patients with thrombocytopenia. Notably, TNBC patients rarely present with severe bone marrow involvement as the initial manifestation. Furthermore, due to its invasiveness, bone marrow aspiration is not a routine diagnostic procedure, and may therefore be missed.

Previous studies suggest that bone marrow metastasis (BMM) is more common in hormone receptor-positive and human epidermal growth factor receptor 2-negative (HR+/HER2−) breast cancer (6, 7). However, in this case, the patient’s breast tumor and bone marrow biopsy specimens were negative for ER, PR, and HER2, contrasting with typical HR-positive BMM presentations. Advanced triple-negative breast cancer (TNBC), characterized by the absence of ER, PR, and HER2 expression, leaves single-agent chemotherapy as a limited treatment option. The patient was a young individual diagnosed with triple-negative breast cancer and bone marrow metastasis. Given the rarity of breast cancer bone marrow metastasis and the absence of specific clinical trials to guide management, we adhered to the treatment principles for advanced triple-negative breast cancer. The OlympiAD trial (8) demonstrated that olaparib significantly prolonged progression-free survival (PFS) compared to chemotherapy, with subgroup analysis suggesting efficacy in patients with visceral and bone metastases. However, BRCA1/2 gene testing in our patient, unfortunately, revealed no relevant mutations, thus substantially weakening the evidence for olaparib use in this case. PD-L1 testing showed a Combined Positive Score (CPS) of 3. As demonstrated by the Keynote-355 trial data (9), no statistically significant difference was observed in the 1≤CPS<10 subgroup (HR=0.94, 95%CI 0.64-1.38), indicating that this population may not benefit from immunotherapy. A CPS of 3 represents a very low level of PD-L1 expression, likely insufficient to activate a robust anti-tumor immune response. Furthermore, considering potential side effects, the multidisciplinary team decided against immunotherapy. In subgroup analyses of the SCENT trial (10), sacituzumab govitecan showed significant survival benefit in patients with extensive metastases beyond brain metastases, including bone metastases. However, due to local policies, Trop-2 ADC drugs are not included in the medical insurance formulary. To avoid exacerbating the patient’s financial burden, the use of Trop-2 ADC drugs was also not considered. Due to severe thrombocytopenia, which chemotherapy could exacerbate, the patient received aggressive transfusion support. Gaurav Pahouja previously reported a patient with severe pancytopenia where daily platelet transfusions combined with doxorubicin controlled bone marrow suppression by the fourth cycle (11). Most studies indicate that patients responding to systemic therapy, such as anthracyclines, taxanes, CDK4/6 inhibitors combined with endocrine therapy, and trastuzumab, may achieve some benefit (7, 11–14). However, not all patients respond. Sasada et al. described a breast cancer patient with BMM who was unresponsive to weekly paclitaxel, transfusions, and G-CSF, eventually dying from gastrointestinal bleeding due to disseminated intravascular coagulation (DIC) and pancytopenia from the metastatic disease (15). In our case, the tumor initially responded to paclitaxel, with subsequent disease progression necessitating a switch to eribulin, which again controlled disease. Several small retrospective studies indicate that aggressive systemic therapy does not significantly increase the risk of cytopenias (6, 7, 16). Despite such challenges, durable disease control is achievable. However, intensive chemotherapy and BMM-related pain compromised the patient’s appetite. In the absence of effective nutritional management, the patient developed severe malnutrition complicated by refeeding syndrome, precluding further chemotherapy. Refeeding syndrome is characterized by metabolic derangements (e.g., electrolyte imbalances, impaired glucose tolerance, and vitamin deficiencies) that occur following the resumption of feeding after prolonged starvation, either orally, enterally, or parenterally. Prevention of refeeding syndrome requires identification of high-risk populations (e.g., prolonged starvation, low BMI), rigorous electrolyte monitoring (phosphorus, potassium, magnesium, and thiamine supplementation), and gradual nutritional support (initiating at 10–20 kcal/kg/day with stepwise caloric increases over 5–7 days). Through multidisciplinary collaboration and individualized adjustments (e.g., cardiac/renal dysfunction considerations), these strategies mitigate the risk of metabolic derangements and ensure therapeutic safety (17). This case underscores the need for effective therapies for TNBC patients with symptomatic bone marrow metastasis and the critical role of early nutritional support. This case highlights the urgent need for effective targeted therapies for TNBC, with early nutritional support during treatment.

The mechanisms involved in breast cancer bone marrow metastases are complex and involve multiple cellular and molecular interactions (18, 19). Following hematogenous spread, breast cancer cells survive and proliferate using the bone marrow microenvironment. Endothelial cells, osteoblasts, and mesenchymal stem cells (MSCs) play a crucial role in the process (20, 21). Cancer cells interact with the bone marrow stroma via signaling molecules such as CXCL12/CXCR4, and by doing so they can occupy the bone marrow niche of hematopoietic stem cells, and then proliferate or become dormant (18). Furthermore, cancer cells disrupt the bone tissue by secreting osteolytic factors such as RANKL and IL-11, as well as releasing growth factors such as TGF-β, creating a self-perpetuating malignant cycle. Marrow adipocytes and adipokines (e.g., leptin and adiponectin) promote cancer cell migration and survival (20). Additionally, cancer cells promote the conversion of MSCs to cancer-associated fibroblasts (CAFs), further enhancing their stem cell-like properties and invasiveness (20, 21).

This case emphasizes that in TNBC patients, particularly those with cytopenia, bone marrow metastasis should be considered. Bone marrow biopsy should be performed for definitive diagnosis, even if conventional imaging is unremarkable. Given the limitations of conventional therapies for TNBC with bone marrow metastasis, emerging treatment modalities such as targeted therapy and immunotherapy should be explored. Multidisciplinary collaboration is also essential to optimize care and treatment outcomes. Patient monitoring and treatment should be tailored to individual responses and drug resistance, to achieve better treatment outcomes.

## Conclusion

This report describes a rare case of TNBC with severe thrombocytopenia as the initial presentation. The case emphasizes the aggressive nature of bone marrow involvement in TNBC and the complexities of its treatment. While paclitaxel monotherapy can be effective in the short term, more effective therapeutic strategies are urgently needed. Furthermore, this case emphasizes the importance of nutrition in the management of patients with advanced cancers. Future studies should focus on investigating the underlying mechanisms of bone marrow involvement in TNBC, developing precision therapy approaches, and optimizing patient management through multidisciplinary care to improve patient outcomes.

## Data Availability

The original contributions presented in the study are included in the article/**Supplementary Material**. Further inquiries can be directed to the corresponding author.

## Supplementary material

Supplementary Figure 1 Microscopic examination of the axillary lymph node specimen. (A) Hematoxylin and eosin staining of the right axillary lymph node biopsy shows metastatic adenocarcinoma (×100); (B) Estrogen receptor negative; (C) Ki67 proliferation index (50%).

Supplementary Figure 2 The status of the tumor after chemotherapy. (A) A 3x3 cm hard mass in the right breast with unclear boundaries, poor mobility, accompanied by local skin swelling and redness; (B) The breast mass on contrast-enhanced MRI has significantly reduced in size compared to previous scans; (C) The liver mass on contrast-enhanced CT has significantly reduced in size compared to previous scans.
