# Supplementary figures and images for: Symptomatic bone marrow metastasis in triple-negative breast cancer: a case report and literature review
Source: Front Oncol. 2025 Mar 28;15:1570355. doi: 10.3389/fonc.2025.1570355 (PMC11985764; doi:10.3389/fonc.2025.1570355)

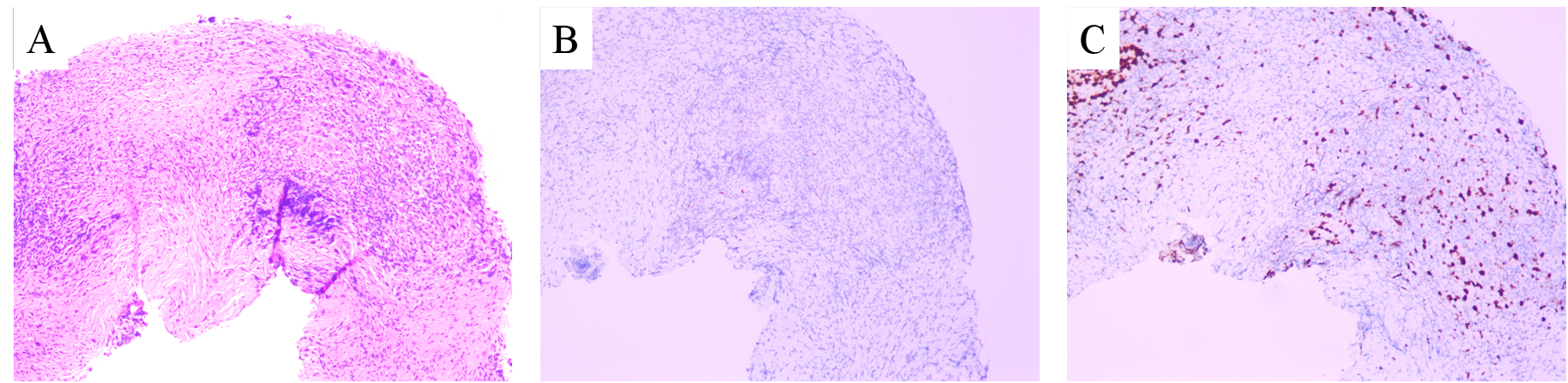

Supplement: Supplementary Figure 1 [file Image1.tif]

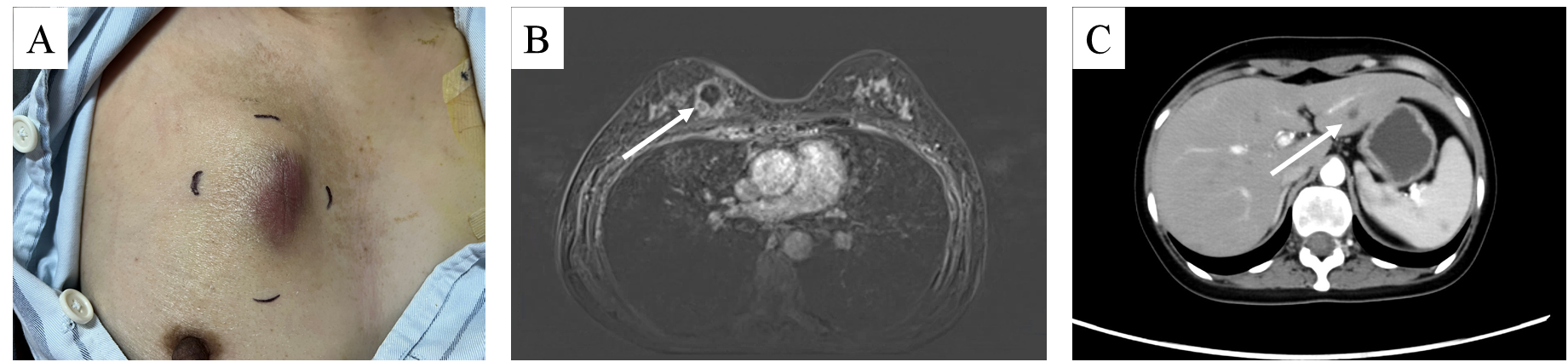

Supplement: Supplementary Figure 2 [file Image2.tif]
